# miR-223-3p carried by cancer-associated fibroblast microvesicles targets *SORBS1* to modulate the progression of gastric cancer

**DOI:** 10.1186/s12935-022-02513-1

**Published:** 2022-02-22

**Authors:** Xiaoli Jin, Xi Qiu, Yi Huang, Hang Zhang, Kaibo Chen

**Affiliations:** 1 Department of Gastrointestinal Surgery, The Second Affiliated Hospital of Zhejiang University School of Medicine, 88 Jiefang Road, Hangzhou, 310009 Zhejiang China; 2 Department of Hematology, The Second Affiliated Hospital of Zhejiang University School of Medicine, Hangzhou, 310009 Zhejiang China

**Keywords:** Cancer-associated fibroblasts, Microvesicles, miR-223-3p, *SORBS1*, Gastric cancer

## Abstract

**Background:**

Cancer-associated fibroblasts (CAFs) aggravate gastric cancer (GC) development.

**Methods:**

Combined with bioinformatics analysis and literature review, miR-223-3p had high expression in microvesicles (MVs) derived from GC CAFs, and it could modulate *SORBS1*. miR-223-3p and *SORBS1* mRNA levels were assessed by qRT-PCR. The levels of CAFs markers, MVs markers, epithelial-mesenchymal transition (EMT)-associated proteins, and *SORBS1* protein were assessed by western blot. MVs isolated from fibroblasts were observed by transmission electron microscopy. Combined with immunofluorescence and co-culture experiments, GC cells were determined to absorb MVs carrying miR-223-3p. Cell functions were measured using CCK-8, transwell, flow cytometry and colony formation assays. The binding of miR-223-3p and *SORBS1* was determined by dual-luciferase assay and RNA immunoprecipitation. The cancer-promoting effect of MVs carrying miR-223-3p on experimental animals was verified in vivo by tumor-bearing experiment in nude mice.

**Results:**

miR-223-3p was upregulated in the MVs secreted by GC CAFs and could be transmitted to GC cells through MVs, to boost the malignant progression of tumor cells. Additionally, it was also revealed that miR-223-3p targeted *SORBS1* and accelerated progression along with EMT in GC.

**Conclusions:**

CAFs-derived MVs could carry miR-223-3p to GC cells to target *SORBS1*, thereby promoting the malignant progression of GC.

## Background

Gastric cancer (GC) is a frequent tumor type across the globe [1]. The treatment conditions for GC have been ameliorated, but patients still face a grim survival status [2]. Therefore, early diagnosis and effective treatment are particularly significant. The study of the pathogenesis of GC can offer a rationale behind new treatment techniques.

Recently, the mechanism of cancer-associated fibroblasts (CAFs) in GC has become one of the research hotspots [3]. In the tumor microenvironment, cancer cells activate non-carcinoma fibroblasts (NFs) by secreting some growth factors and induce them to transform into CAFs. In turn, CAFs also secrete more growth factors to promote the malignant progression of cancer cells [4]. Microvesicles (MVs) are one of the important substances secreted by CAFs. MVs are some vesicles that are detached from cells during physiological or pathological processes. Their size is generally 100–1000 nm. They can carry nucleic acids, lipids and proteins, and deliver them to nearby or distal cells to affect the function and behavior of the recipient cells. MVs can promote cancer cell angiogenesis, drug resistance, proliferation and metastasis in different cancer types by transmitting growth factors, signaling molecules, DNA, coding and non-coding RNA, etc [5–7]. Hence, it is of great meaning to study the specific regulatory effect of MVs in GC CAFs on cancer cells.

MicroRNAs (miRNAs) can bind to mRNAs to achieve post-transcriptional regulation in cells, thereby affecting a variety of cell biological functions [8]. miR-223-3p can modulate solid tumor development. For example, miR-223-3p boosts the malignant progression of prostate cancer by targeting SEPT6 [9]. miR-223-3p suppresses glioma cell proliferation via targeting inflammation-related cytokines [10]. Meanwhile, one study revealed that miR-223-3p can also aggravate the malignant progression of GC [11].

Sorbin and SH3 domain-containing protein 1, also CAP/ponsin (*SORBS1*) is an adaptin in nature [12] that interacts with cytoskeleton regulators to mediate cytoskeleton structural organization [13], and cell spreading and movement [14]. *SORBS1* can also hamper tumor metastasis and enhance the sensitivity of cancer to chemotherapy drugs [15]. Based on the existing research background, we designed a series of experiments to further relevant mechanisms. Through experiments, it was uncovered that CAFs-secreted MVs carried miR-223-3p in GC tissue targeted *SORBS1*, and played a cancer promotor role. This study has practical implications for future GC treatment and drug development.

## Materials and methods

### Bioinformatics methods

GSE93415 dataset (cancer tissue (CT): 20; corresponding para-cancerous tissue (PT): 20) of GC tissue was offered by the Gene Expression Omnibus (GEO) database. R package limma was applied for differential analysis for miRNAs (padj < 0.05, |logFC|> 1.5). Combined with the literature, the target miRNA (miR-223-3p) was determined. Then, miR-223-3p expression in tissue, cells, tissue fluid, blood exosomes and MVs was predicted using EVmiRNA database. The targets of miR-223-3p were predicted using miRDB, mirDIP, and Targetscan tools. The Cancer Genome Atlas (TCGA)-STAD data were obtained, and edgeR package was utilized for differential analysis (|logFC|> 2, padj < 0.05) to acquire differentially expressed mRNAs (DEmRNAs). The predicted target genes were overlapped with the DEmRNAs to determine the mRNA targeted by miR-223-3p.

### Clinical samples

In this study, 20 cases of CT samples and 20 corresponding PT samples (more than 10 cm away from the negative margin) were gathered from GC patients in The Second Affiliated Hospital of Zhejiang University School of Medicine from 2018 to 2020. Each CT and paired PT samples were from the same patient. Part of the tissue samples were collected for the isolation of fibroblasts, and the other part were stored at − 80 ℃ until use. All cases were assessed by pathologists based on histopathology, re-checked by pathologists and defined with GC (American Joint Committee on Cancer Version II, III, IV, VII). This study got approval from the Ethics Committee of The Second Affiliated Hospital of Zhejiang University School of Medicine with written informed consent from all participants.

### Extraction of CAFs, NFs and tumor cells (TCs)

Fresh CT or PT samples were rinsed with Dulbecco’s Modified Eagle medium (DMEM) (Thermo Fisher Scientific, USA) without serum, and transferred to 0.15% collagenase IV (Thermo Fisher Scientific, USA) solution. Then at 37 °C, they were cultivated (Thermo Fisher Scientific, USA) with 5% CO_2_ for 40 min. The fully digested cells were filtered through a 40 mm cell filter (BD Biosciences, USA) and centrifuged for 10 min (1500 rpm). Cell suspension was cultured in fibroblast medium (Innoprot, Spain) for 24 h for adherent growth. After 24 h, the nonadherent cells were washed off and the adherent ones were subcultured further. The fibroblasts isolated from GC tissue were used as CAFs, and the fibroblasts isolated from adjacent tissue were used as NFs. TCs were isolated from fresh GC tissue using the Cancer Cell Isolation Kit (Thermo Fisher Scientific, USA).

### Cell culture

Human GC cell line SGC7901 (GDC150) was acquired from China Center for Type Culture Collection (CCTCC). Human GC cell lines AGS (BNCC309318) and BGC-823 (BNCC337689), and normal gastric mucosal cell line GES-1 (BNCC337970) were offered by BeNa Culture Collection (China). The above cell lines were cultivated in Roswell Park Memorial Institute (RPMI) 1640 medium (Media, USA) containing 10% fetal bovine serum (FBS) (Thermo Fisher Scientific, USA) and cultured at 37 °C with 5% CO_2_ (Thermo Fisher Scientific, USA). CAFs and NFs isolated from tissue samples were cultured in fibroblast medium (Innoprot, SPIAN) containing 10% FBS (Thermo Fisher Scientific, USA) under routine conditions.

### Cell transfection

miR-223-3p mimic, miR-223-3p inhibitor, si-*SORBS1* and corresponding negative controls (NCs) were designed and provided by Shanghai GenePharma Co. Ltd. (China). Cell transfection was undertaken using Lipofectamine 3000 (Thermo Fisher Scientific, USA).

### Extraction of MVs

MVs were extracted from cell culture medium by hypervelocity centrifugation. Briefly, the supernatant of the cell medium was first taken, and the debris was removed by centrifugation at 3000×*g* for 10 min. Then a 100,000×*g* centrifuge was conducted at 4 °C for 2 h using an ultracentrifuge (Thermo Fisher Scientific, USA). Finally, the extracted MVs were resuspended in phosphate buffer saline (PBS) to prepare for further transmission electron microscope (TEM) observation. The concentration of MVs was evaluated by bicinchoninic acid assay (BCA, Thermo Scientific, USA).

### TEM

The MV suspension was loaded into a carbon film-coated TEM copper grid, stained with uranyl acetate, and then dried. MVs were observed and photographed using Hitachi JEM-2100 TEM (Japanese Electronics Co., Ltd., Tokyo, Japan).

### Co-culture of MVs and GC cells

5 × 10^5^ GC cells were inoculated in 25-cm^2^ culture dish. 100 ug MVs were added to 5 ml complete medium. 24 h later, GC cells were gathered for the following steps. MVs co-culture was conducted as the MV treatment group in the cell experiment.

### qRT-PCR

RNA was isolated using the miRNeasy mini kit (Qiagen, Germany). The purity and concentration of the extracted RNA were detected by NanoDrop 2000. The corresponding complementary DNA (cDNA) was obtained by reverse transcription with miScript II RT (Qiagen, Germany). The expression level was ascertained through qRT-PCR by using miScript SYBR Green PCR Master Mix (Qiagen, Germany). The qPCR detection was performed using Real-Time PCR on ABI7500 (Thermo Fisher Scientific, USA). U6 and GAPDH were selected as internal references. All PCR primers were designed by Shanghai Genepharma Co, Ltd. (China) as displayed in Table 1.

**Table 1:** qRT-PCR primer sequence

Gene	Primer sequence (5’ → 3’)
miR-223-3p	F: AGCTGGTGTTGTGAATCAGGCCG
	R: TGGTGTCGTGGAGTCG
SORBS1	F: ATTCCCAAGCCTTTCCATCAG
	R: TTTTGCTGTTCTCGATTGTGTTG
U6	F: CTCGCTTCGGCAGCACA
	R: AACGCTTCACGAATTTGCGT
GAPDH	F: GAACGGGAAGCTCACTGG
	R: GCCTGCTTCACCACCTTCT

### Western blot

MVs and cells were cleaved in radioimmunoprecipitation assay (RIPA) cell lysis reagent containing the protease inhibitor. The equivalent protein samples (15 μg) were electrophoresed on 10% sodium dodecyl sulfate polyacrylamide gel electrophoresis gel, and then mounted on polyvinylidene fluoride (Bio-Rad Laboratories, Inc., USA). After SDS-PAGE, the membrane was blocked with 5% skimmed milk at room temperature for 1 h and then incubated overnight with the primary antibodies at 4 °C. The primary antibodies were rabbit anti-human antibodies: anti-α-SMA antibody (ab5694, Abcam, UK), anti-FAP antibody (ab207178, Abcam, UK), anti-CEA antibody (ab207718, Abcam, UK), anti-CK-18 antibody (ab133263, Abcam, UK), anti-CD63 antibody (ab134045, Abcam, UK), anti-E-cadherin antibody (ab40772, Abcam, UK), N-cadherin antibody (ab76011, Abcam, UK), anti-vimentin antibody (ab92547, Abcam, UK), anti-*SORBS1* antibody (ab224129, Abcam, UK), and anti-GAPDH antibody (ab6721, Abcam, UK). The secondary antibody was horseradish peroxidase-labeled goat antirabbit IgG antibody (ab6721, Abcam, UK).

### CCK-8 assay

The pretreated GC cells were inoculated into 96-well plates (5 × 10^3^ per well) and cultured for 0, 24, 48, and 72 h, respectively. Afterward, 10 μl CCK-8 solution was added at each period (MedChem Express, USA). The absorbance at 450 nm was tested with a microplate analyzer.

### Colony formation assay

The pretreated GC cells (5 × 10^2^ per well) were plated into 12-well plates and incubated in cell medium with 10% FBS for two weeks. After completion of culture, the cell colonies were fixed with 10% formaldehyde and stained using crystal violet dye. The staining results were photographed and then analyzed using Image J software.

### Transwell assay

For invasion assay, 50 μl matrix gel U5GL (BD Biosciences, USA) was applied to the upper chamber. The pretreated GC cells (2 × 10^4^ per well) were suspended in 100 μl medium without serum and inoculated in the upper chamber (BD Biosciences). The lower chamber was added with 500 μl medium containing 20% FBS. After 48 h incubation, the uninvaded cells were wiped with cotton swabs, and the remaining cells were fixed with 4% polyformaldehyde and stained using 0.1% crystal violet for 15 min. Then, 5 random visual fields per chamber were selected for counting cells under a microscope (100 ×). The cell migration experiment was similar to the above steps, except for the following differences: (1) no matrix gel coating was required at the upper surface of the insert; (2) 24 h of incubation.

### Dual-luciferase reporter gene detection

Firstly, the targeting sequence of miR-223-3p in *SORBS1* 3’- untranslated region (UTR) was predicted by miRDB. The wild-type (WT) and mutant (MUT) *SORBS1* 3’-UTR were then amplified by PCR and introduced into the pMIR-REPORT vectors (AddGene, USA). pRL-TK plasmid was the internal reference luciferase reporter plasmid (AddGene, USA). Finally, miR-223-3p or corresponding NC and constructed reporter gene plasmid were co-transfected into GC cells. After 48 h, fluorescence activity was evaluated on the Dual-Luciferase® Reporter Assay System.

### Cell uptake of MVs

MVs were stained using PHK-26 (Umibio, China). The PHK-26 labeled MVs were co-incubated with GC cells for 24 h under general conditions. A fluorescence microscope (Nikon, Japan) was employed to observe the initial imaging and imaging after 0, 6, and 24 h. Flow cytometer was utilized for analyses and the data acquired were subject to calculation of median fluorescence intensity (MFI) of GC cells (MVs carrying MVs).

### Xenograft mouse models

GC cells (2 × 10^6^ cells/ml) were injected into the lower left limb of 10 BALB/c nude mice (Laboratory Animal Resources, Chinese Academy of Sciences, China) aged 4–6 weeks. After 3 days, tumor mass formation was observed. On day 8, nude mice were divided into 3 groups randomly (PBS group; inhibitor NC group and inhibitor group, 5 mice in each group), and MVs (miR-223-3p inhibitor and its NC were respectively transfected into CAFs and then MVs were isolated. PBS was added to attenuate the MV suspension with a protein concentration of 0.5 ug/ul. 15 μg/mouse) or corresponding volume of PBS were injected into tumor mass of nude mice in different groups (marked as day 0). The injection was done every 3 days, before which MV suspension was blown and mixed evenly. The mass size was measured with a vernier caliper every 3 days according to formula 1/2 (length × width^2^). All the mice were euthanized by carbon dioxide asphyxiation on day 24, and death was confirmed after 5 min of observation. When performing euthanasia using CO_2_ asphyxiation, the mice were in the induction phase and provided with air. The CO_2_ concentration was constantly increased until the mice were confirmed by cardiac arrest and respiratory arrest. Besides, the CO_2_ was at a flow displacement rate of 30%-70% of the chamber volume per min, so as to ensure that the mice were unconscious prior to pain. The tumor masses were removed and weighed. The tumor masses were also used for subsequent qRT-PCR detection and immunohistochemical (IHC) assay.

### IHC assay

The paraffin-embedded tissue fixed with formaldehyde was sectioned (4 um), and then the tissue was placed in an oven at 60 °C and heated for 30 min before being dewaxed with xylene. The endogenous peroxidase was then removed by incubation with 3% H_2_O_2_ for 10 min. After blocking, the sections were added with primary anti-Ki67 (ab15580, Abcam, UK) to incubate overnight at 4 ℃. On the next day, after being washed by PBS, sections were reacted with secondary antibody IgG H&L (HRP) at 37 ℃ for 1 h, and then colored by DAB (3,3’-diaminobenzidine). The staining was observed with an upright optical microscope.

### RNA immunoprecipitation (RIP)

This step was taken using RNA binding protein immunoprecipitation kit (Millipore, Bedford, MA). miR-223-3p mimic-treated GC cells were lysed with RIPA buffer (Cell Signaling Technology) containing phosphatase and proteases inhibitors (Sigma-Aldrich). Magnetic beads (Invitrogen) were incubated with IgG antibody (Cell Signaling Technology) or Ago2 for 30 min. The lysate was subjected to immunoprecipitation and rotated at 4 °C. RNA was purified from RNA–protein complex binding beads for qRT-PCR analysis.

### Flow cytometry

Pre-treated cells were rinsed with cold PBS and fixed with 80% ethanol. The cells were then centrifuged in a cold spinning machine and re-suspended in cold PBS. Being cultured at common temperature for 30 min, cells were added with propidium iodide (Sigma-Aldrich; 20 mg/ml) and bovine pancreatic RNAase (Sigma; 2 mg/ml) for 20 min of incubation. 2 × 10^4^ cells were analyzed using BD FACSCanto, the data of which were assessed using FLOWJO software (Tree Star, Inc, Ashland, OR).

### TdT-mediated dUTP-biotin nick end labeling (TUNEL) technique

Cell apoptosis detection kit (Roche, USA) was utilized for TUNEL. Paraffin-embedded sections were subjected to gradient hydration, fixation with 4% formaldehyde, and incubation with protease K at common temperature for 15 min. 3% hydrogen peroxide was utilized to block endogenous peroxidase. Fresh TUNEL reaction solution containing rTdT was prepared. The sections were washed with PBS and counterstained with hematoxylin. Apoptotic cells were tested with a microscope (Nikon, Japan).

### Data analysis

Data processing was performed using GraphPad Prism (GraphPad Software, USA). All assays were performed at least 3 times and the results were exhibited as mean ± standard deviation. One-way analysis of variance was used to compare the differences between groups, and *t*-test was used for post hoc test. *P* < 0.05 represented statistically remarkable, and * in the figure denotes *p* < 0.05.

## Results

### The expression of miR-223-3p is upregulated in GC and MVs of CAFs

**Fig. 1:**
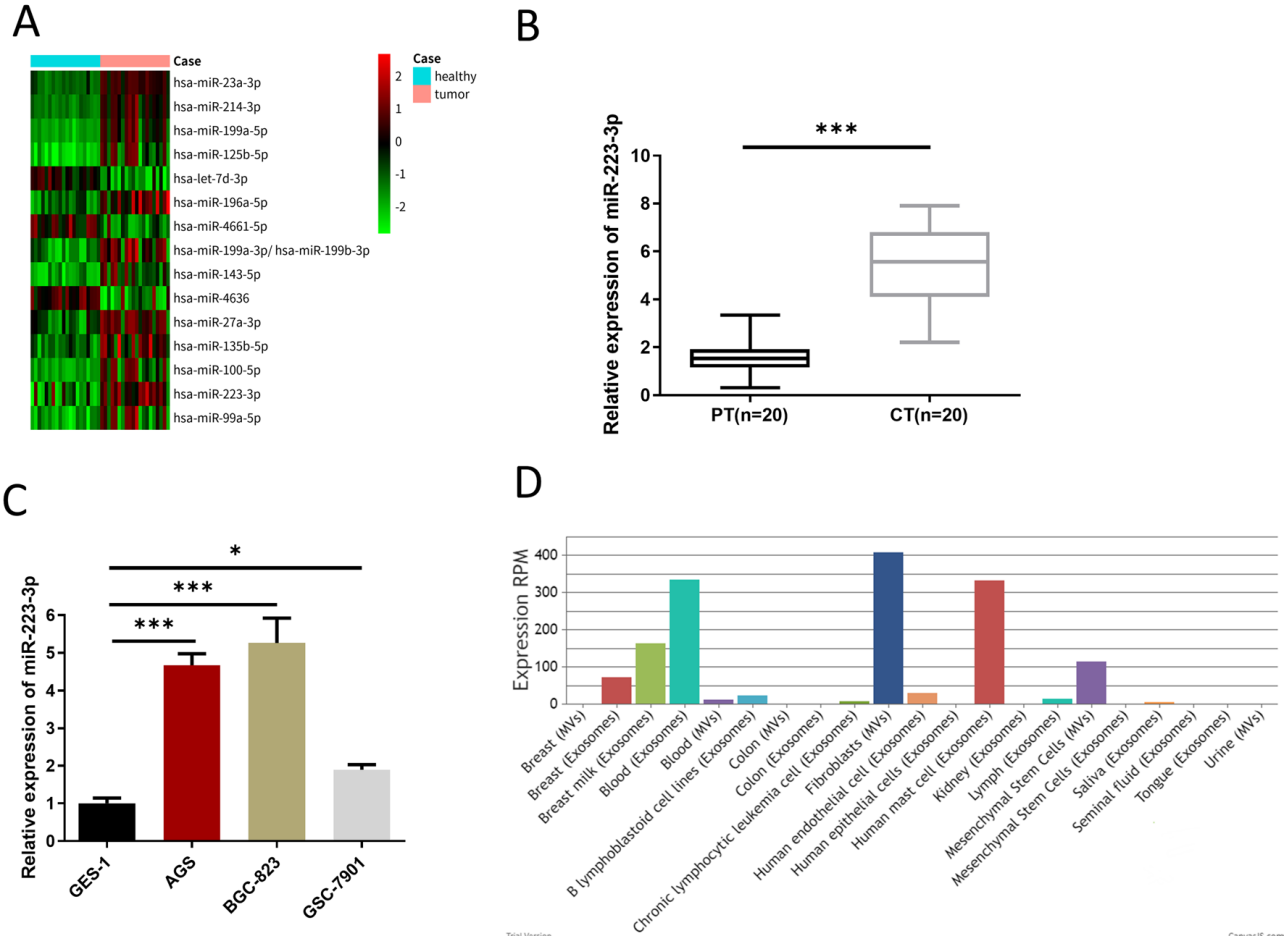
The expression of miR-223-3p is upregulated in GC and MVs of CAFs. **A** The expression of 15 different miRNAs in 20 GC samples retrieved from GEO database; **B** Expression of miR-223-3p in PT and CT (n = 20, *p* < 0.001); **C** Expression of miR-223-3p in the normal gastric mucosa cell line (GES-1) and GC cell lines (AGS, BGC-823, GSC-7901) at logarithmic phase (n = 3, *p* < 0.001; *p* < 0.001; *p* = 0.0496); **D** The expression of miR-223-3p in different tissue, cells, tissue fluid, blood exosomes and MVs retrieved from the EVmiRNA database. * *p* < 0.05; *** *p* < 0.001

Limma differential analysis of 20 GC samples in GSE93415 chip was conducted through GEO database. Totally 15 DEmiRNAs were acquired including 12 upregulated genes and 3 downregulated ones (Fig. 1A). Combined with an existing report, miR-223-3p is upregulated in GC, inhibits apoptosis, and enhances the invasion of GC cells [16]. qRT-PCR result exhibited that miR-223-3p level in CT tissue was prominently higher than that in PT tissue (Fig. 1B). Next, qRT-PCR result revealed that the relative expression level of miR-223-3p was the highest in BGC-823 cells (Fig. 1C). Hence, we selected BGC-823 as the experimental GC cell line in the following cell experiments. Finally, miR-223-3p expression in various tissues, cells, tissue fluid, blood exosomes and MVs was searched through the EVmiRNA database, finding that miR-223-3p mostly existed in fibroblast MVs (Fig. 1D). According to the above findings, high expression of miR-223-3p was determined in GC, and it may be mainly derived from MVs secreted by CAFs in GC tissue.

### Increased expression of miR-223-3p in MVs derived from CAFs

**Fig. 2:**
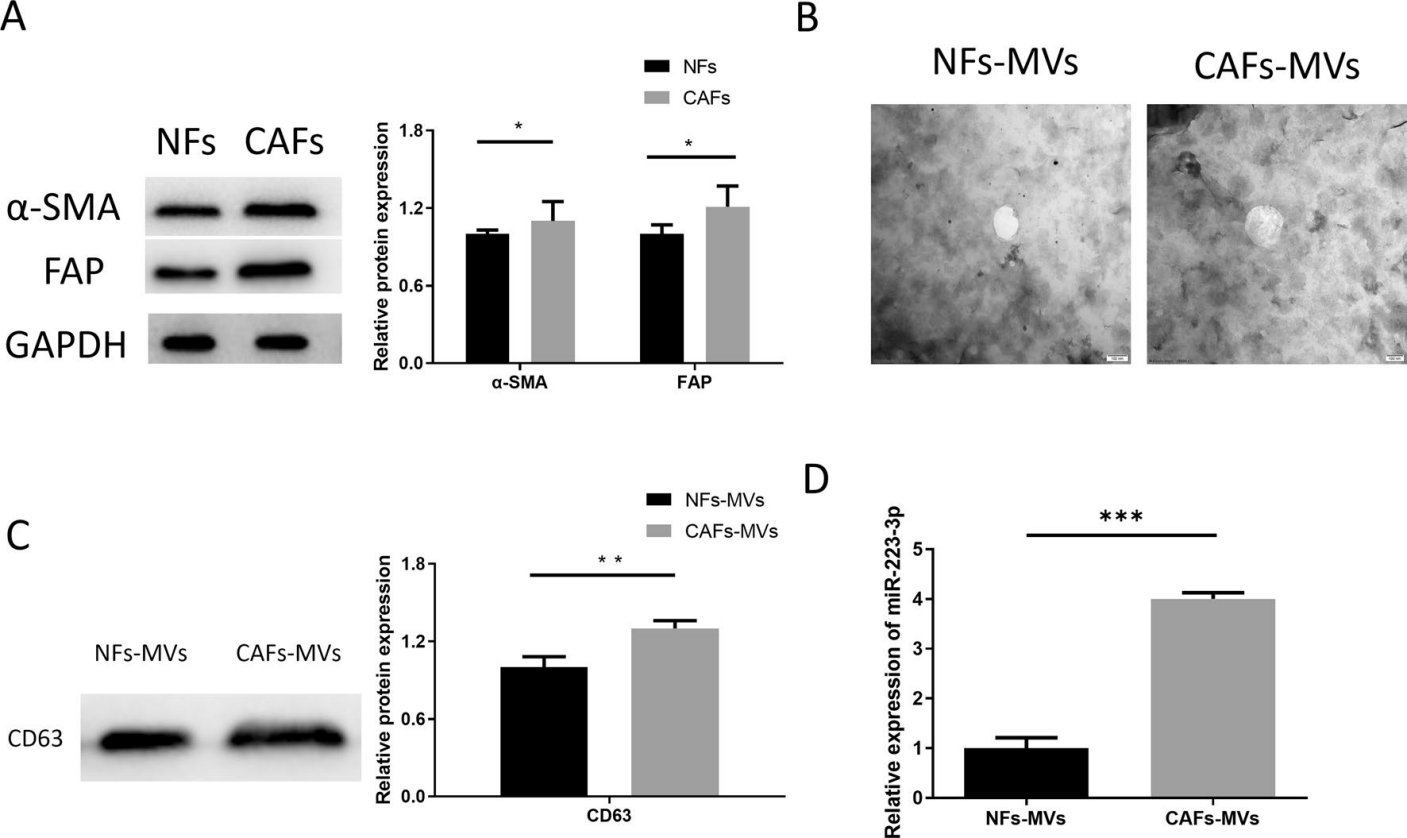
miR-223-3p expression is upregulated in MVs derived from CAFs. **A** Detection of fibroblast marker proteins (α-SMA, FAP) in CAFs, NFs (at logarithmic phase) by western blot (n = 3, *p* = 0.0154; *p* = 0.0186); **B** NFs-MVs and CAFs-MVs observed under TEM with a scale of 100 nm; **C** MV marker protein CD63 in NFs-MVs and CAFs-MVs detected by western blot (n = 3, *p* = 0.065); **D** miR-223-3p expression in CAFs-MVs and NFs-MVs (n = 3, *p* < 0.001). *p < 0.05; **p < 0.01; ***p < 0.001

To confirm whether miR-223-3p is mainly derived from MVs secreted by CAFs in GC tissue, the following experiments were designed. CAFs were isolated from CT of GC patients, while NFs were isolated from PT of GC patients. Then, fibroblast marker proteins (α-SMA, FAP) were measured by western blot (Fig. 2A). The result revealed that fibroblast marker proteins were the most obvious in the CAFs group, indicating that CAFs and NFs were successfully isolated from the CT and PT. Additionally, we first isolated MVs from CAFs and NFs (CAFs-MVs, NFs-MVs), and characterized the physical characteristics of the isolated MVs by TEM (Fig. 2B). At the same time, the MVs marker CD63 was detected by western blot (Fig. 2C). It could be seen that we successfully isolated MVs from NFs and CAFs. Subsequently, qRT-PCR result indicated that miR-223-3p level in CAFs-MVs was remarkably higher than that in NFs-MVs (Fig. 2D). In conclusion, miR-223-3p expressed highly in the MVs secreted by CAFs.

### CAFs- MVs-derived miR-223-3p accelerates the malignant progression of GC

**Fig. 3:**
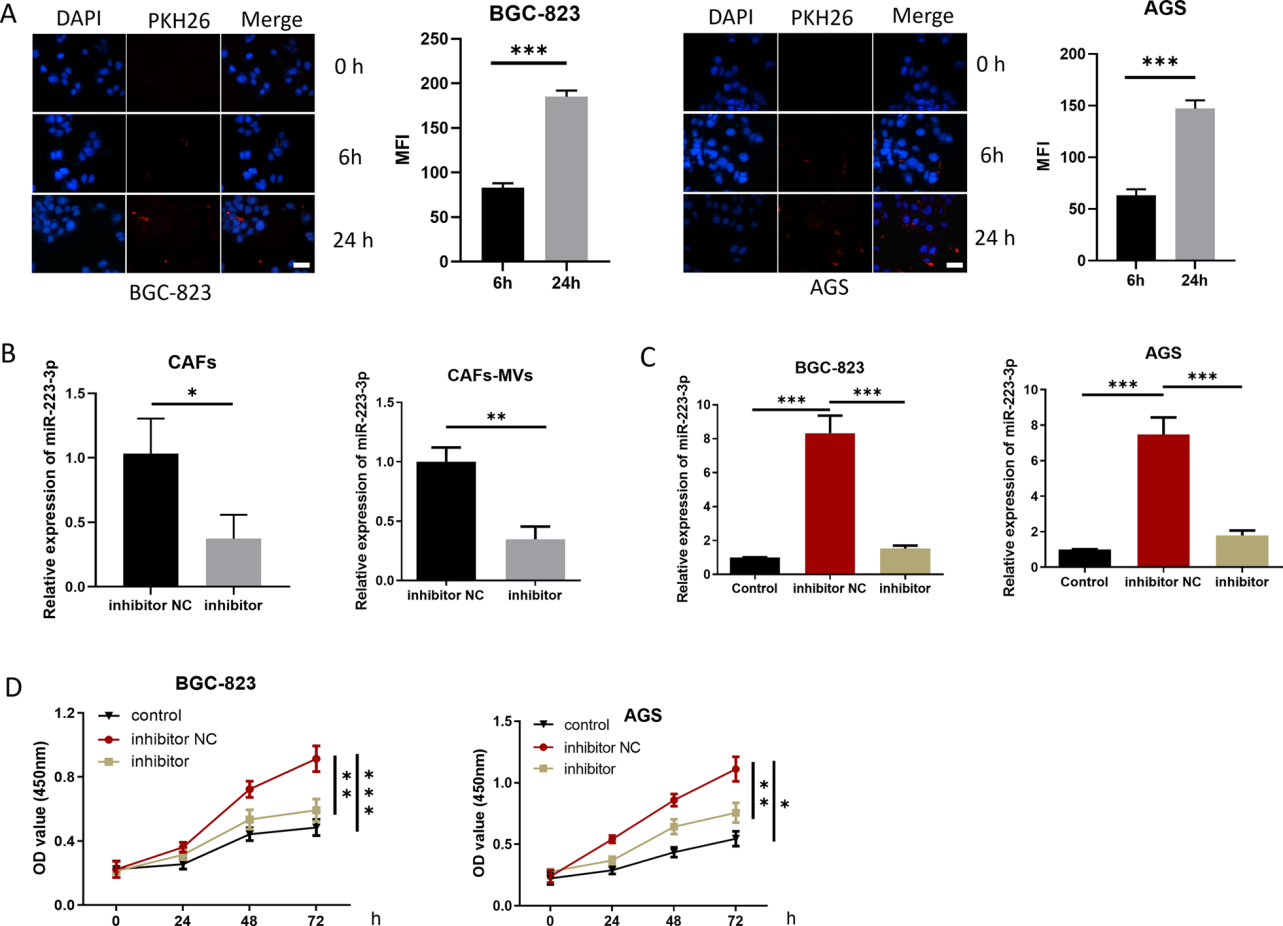

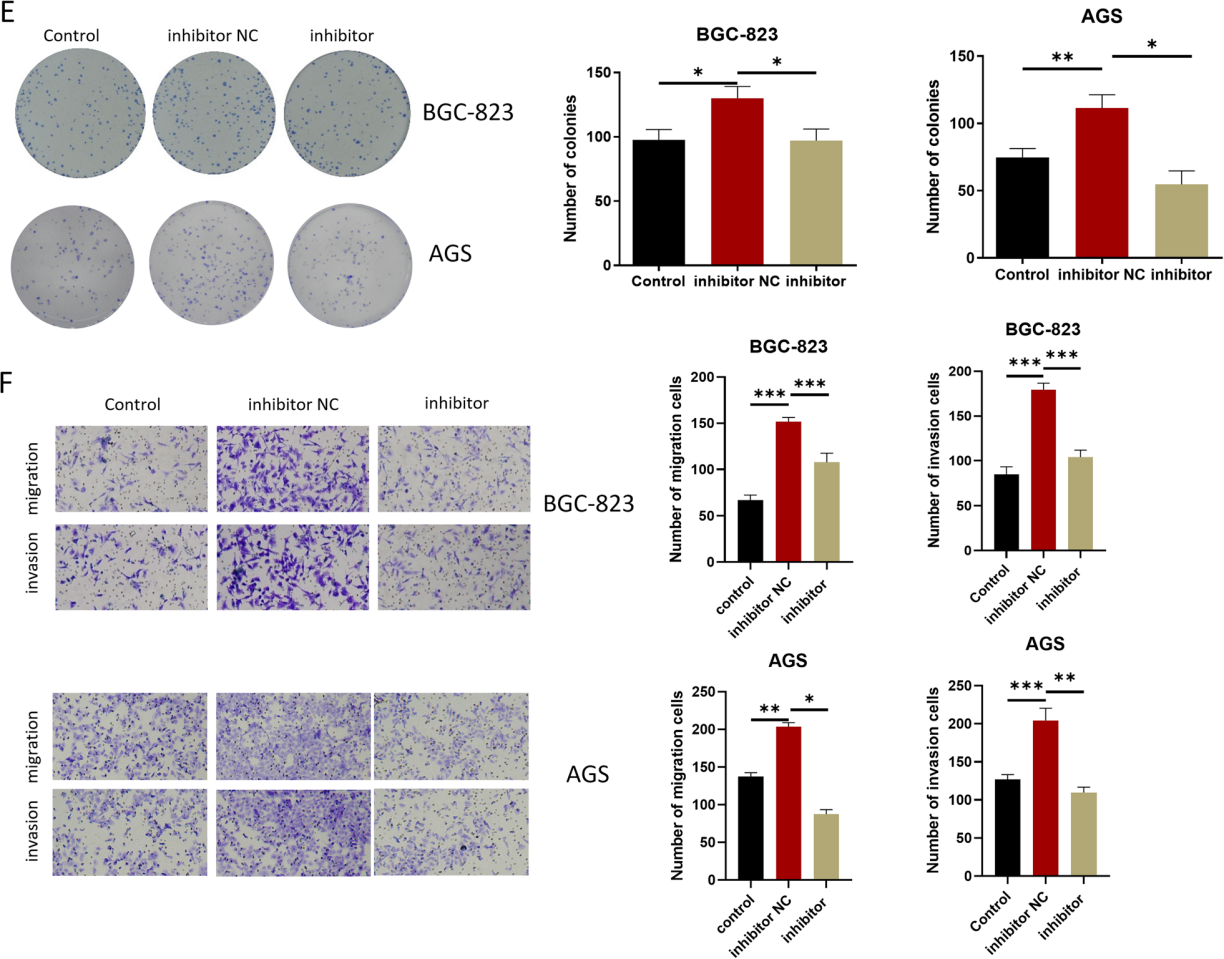

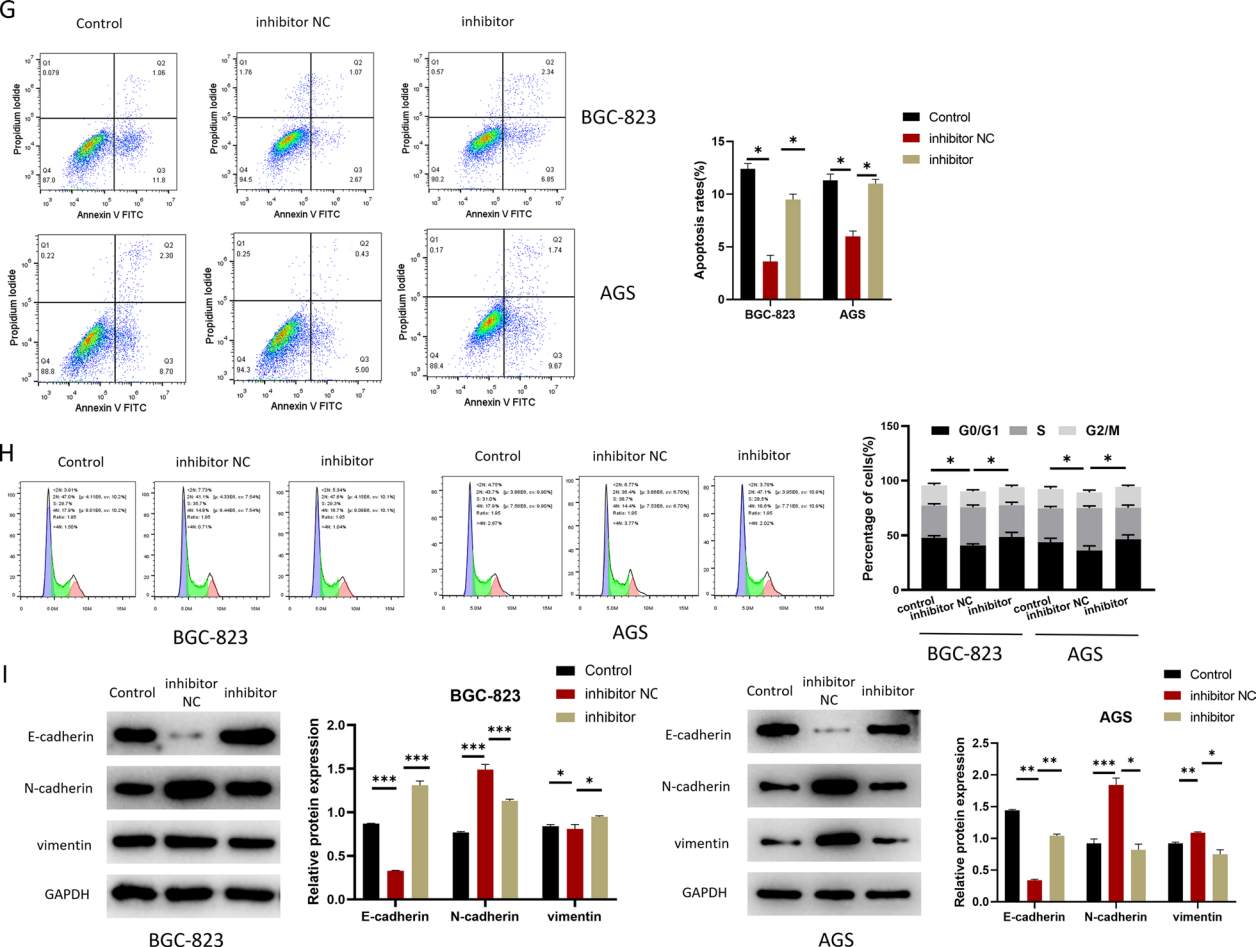
CAFs-MVs-derived miR-223-3p can facilitate malignant progression of GC. **A** PKH26-labeled MVs co-incubated with GC cells (PKH26 in red while DAPI in blue) (n = 3, *p* < 0.0001; *p* < 0.001; scale bar: 20 μm); **B** miR-223-3p level in the CAFs (at logarithmic phase, n = 3, *p* = 0.0237) and MVs of CAFs (n = 3, *p* = 0.0021) in each transfection group (inhibitor NC, inhibitor); **C** miR-223-3p level in GC cells after 24 h of MVs co-cultured with GC cells (n = 3; *p* < 0.001; *p* < 0.001, n = 3; *p* = 0.0003; *p* = 0.0006); **D** The activity of GC cells after co-culture with MVs in each transfected group (control, inhibitor NC, inhibitor) (n = 3; *p* < 0.001; *p* = 0.0028, n = 3; *p* = 0.0011; *p* = 0.0214); **E** The colony-forming ability of GC cells after co-culture with MVs in each transfection group (n = 3; *p* = 0.0125; *p* = 0.0459, n = 3; *p* = 0.0062; *p* = 0.0023); **F** The migration and invasion of GC cells after co-culture with MVs in each transfected group (BGC-823 cells: n = 3; *p* < 0.001; *p* < 0.001, n = 3; *p* = 0.0001; *p* = 0.0003, AGS cells: n = 3; *p* = 0.0001; *p* < 0.0001, n = 3; *p* = 0.0015; *p* = 0.0007); **G** Cell apoptosis status (n = 3; *p* < 0.0001; *p* = 0.0002, n = 3; *p* = 0.0003; *p* = 0.0002); **H** Cell cycle status (n = 3; *p* = 0.0106; *p* = 0.0350, n = 3; *p* = 0.0101; *p* = 0.0224); **I** The expression of EMT-related proteins in GC cells after co-culture with MVs 24 h in each transfected group (BGC-823 cells: n = 3; *p* < 0.0001; *p* < 0.0001, *p* < 0.0001; *p* = 0.0006, *p* = 0030; *p* = 0007, AGS cells: *p* < 0.0001; *p* < 0.0001, *p* = 0.0003; *p* = 0.0002, *p* < 0.0001; *p* < 0.0001). *p < 0.05; **p < 0.01; ***p < 0.001

The following experiments were designed to study cell progression and EMT. First, to confirm whether CAFs-MVs can be absorbed by GC cells, fluorescent marker PKH-26 was used to label CAFs-MVs, and the labeled MVs were co-incubated with GC cells (BGC-823) for 6 and 24 h. MVs were absorbed by the GC cells 6 h later and the increase of MFI in GC cells was more conspicuous 24 h later, suggesting that MVs were effectively absorbed by the GC cells (Fig. 3A). Next, miR-223-3p inhibitor or inhibitor NC was transfected into CAFs and the transfection efficiency was measured by qRT-PCR (Fig. 3B). As qRT-PCR detected, miR-223-3p level in MVs in the inhibitor group was prominently downregulated (Fig. 3B). To study the biological function of miR-223-3p derived from CAFs-MVs on GC cells in vitro, MVs isolated from transfection groups were co-incubated with GC cells (BGC-823 and AGS). The treatment groups were divided into the Control group without MVs treatment, the inhibitor group with CAFs-MVs treatment with miR-223-3p knockdown, and inhibitor NC group with normal CAFs-MVs treatment (Fig. 3C–I). Firstly, qRT-PCR result expressed that miR-223-3p level in the inhibitor NC group was markedly higher than that in the control group. miR-223-3p expression in the inhibitor group was evidently lower than that in the inhibitor NC group and was similar to that in the control group, suggesting that CAFs-MVs could prominently increase miR-223-3p level in GC cells (Fig. 3C). CCK-8 and colony formation assays uncovered that the knockdown of miR-223-3p in CAFs reduced the proliferation-facilitating ability of MVs on GC cells (Fig. 3D, E). Besides, transwell assay observed that miR-223-3p knockdown in CAFs also prominently downregulated the migration and invasion-promoting abilities of MVs in GC cells (Fig. 3F). miR-223-3p knockdown in CAFs conspicuously increased cancer cell apoptotic rate and the cell proportion in G0/G1 phase (Fig. 3G, H). Upregulating N-cadherin and Vimentin along with downregulating E-cadherin are EMT markers, which are critical driver factors for tumorigenesis [17]. The expression E-cadherin, N-cadherin and Vimentin in each group was measured by western blot. The results uncovered that E-cadherin level in the inhibitor NC group was downregulated, but N-cadherin and vimentin expression was upregulated in comparison with the control group. Compared with the inhibitor NC group, E-cadherin level in the inhibitor group was elevated, but the expression of N-cadherin and vimentin was decreased (Fig. 3I). The results suggested that the impact of miR-223-3p knockdown on the promotion of EMT in GC cells was inhibited. Combined with the above results, CAFs-MVs carrying miR-223-3p boosted the progression and EMT, and modulated cell cycle and apoptosis of GC cells.

### miR-223-3p targets and regulates *SORBS1*

**Fig. 4:**
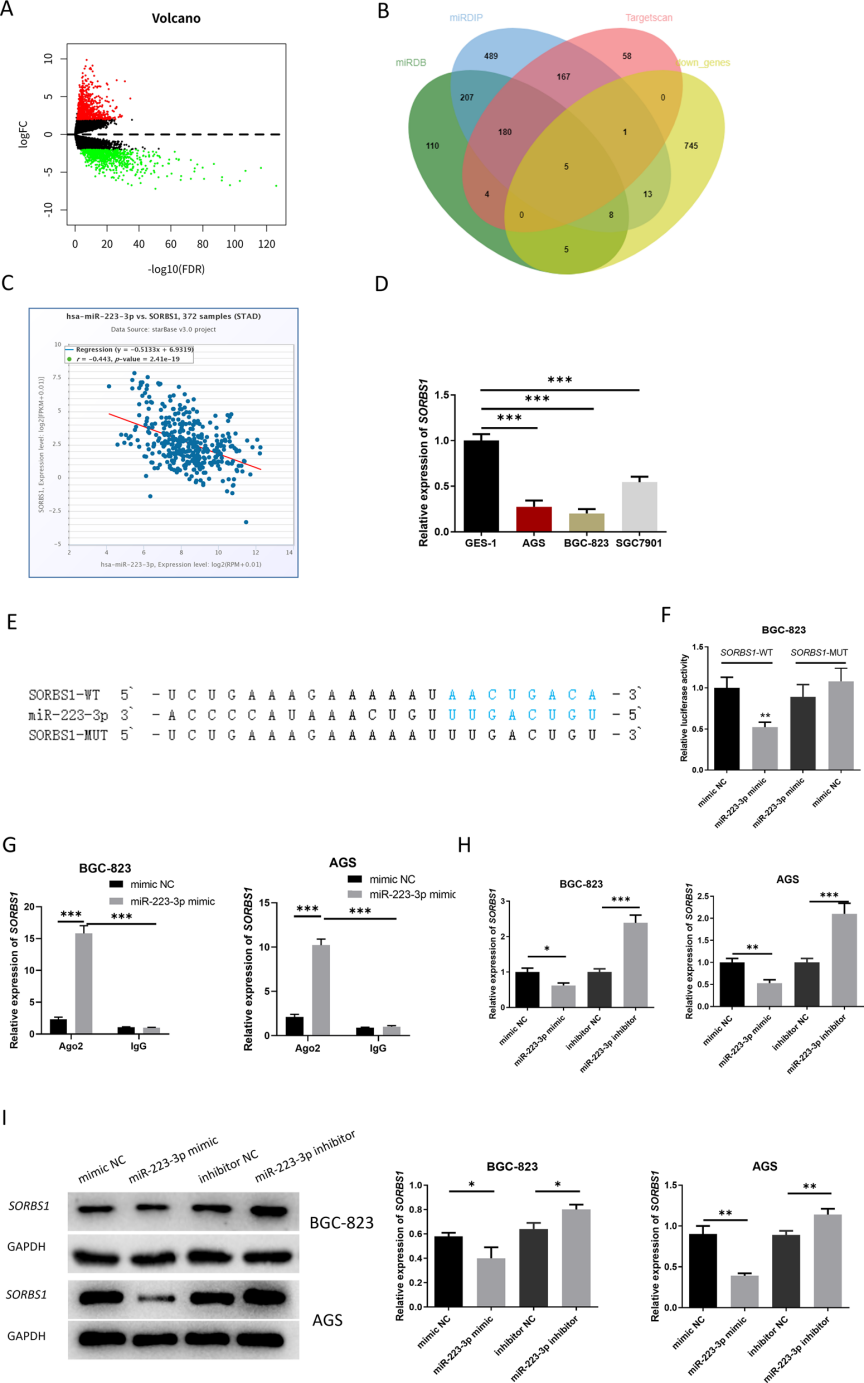
miR-223-3p targets and modulates *SORBS1*. **A** Differential expression of GC-related mRNA by analysis in TCGA-STAD; **B** The intersection between results predicted by 3 databases (Targetscan, miRDB, mirDIP) and the differential genes; **C** The correlation between miR-223-3p and potential target genes analyzed by ENCORI (*p* = 2.41e−19); **D** *SORBS1* expression in normal gastric mucosa cells and GC cells (at logarithmic phase) (n = 3; *p* = 0.0002; *p* < 0.0001; *p* = 0.001); **E** The binding sites of miR-223-3p and *SORBS1* predicted by miRDB database; **F** Targeted relationship between miR-223-3p and *SORBS1* verified by dual-luciferase reporter gene detection (n = 3; *p* = 0.0092); **G** Interaction between miR-223-3p and *SORBS1* verified by RIP (n = 3; *p* < 0.0001; *p* < 0.0001, n = 3; *p* < 0.0001; *p* < 0.0001); **H** *SORBS1* level in each transfection group after 48-h transfection (n = 3; *p* = 0.0367; *p* < 0.001, n = 3; *p* = 0.0024; *p* = 0.0017); **I** The protein expression level of *SORBS1* in each transfection group after 48-h transfection (n = 3; *p* = 0.0303; *p* = 0.0124, n = 3; *p* = 0.0011; *p* = 0.0073). *p < 0.05; **p < 0.01; ***p < 0.001

We conducted differential analysis of GC-related mRNA data through TCGA-STAD, and obtained 1679 DEmRNAs, 777 of which were downregulated and 902 were upregulated (Fig. 4A). Subsequently, the intersection of related downregulated genes was obtained from 3 databases (Targetscan, miRDB, mirDIP) to obtain 5 downregulated targets of miR-223-3p (Fig. 4B). Correlation analysis was conducted by using ENCORI database, uncovering that the negative correlation between *SORBS1* and miR-223-3p was the most obvious (Fig. 4C). Based on the above bioinformatics results, qRT-PCR result exhibited that *SORBS1* expression in GC cell lines was evidently lower than that in the normal gastric mucosa cell line (Fig. 4D). *SORBS1* was a potential underlying target of miR-223-3p as predicted by miRDB database. The predicted result expressed that the two had potential binding sequences (Fig. 4E). The targeted relationship between the two was verified by dual-luciferase detection. The result revealed that miR-223-3p mimic remarkably reduced the fluorescence activity of cells with *SORBS1*-WT 3’-UTR but failed to change that of cells with *SORBS1*-MUT 3’-UTR (Fig. 4F). It was suggested that miR-223-3p targeted the 3’-UTR of *SORBS1*. Further qRT-PCR results indicated that *SORBS1* mRNA and protein expression levels were markedly reduced in the miR-223-3p mimic group but increased in the miR-223-3p inhibitor group (Fig. 4G–I). According to the above analyses, miR-223-3p negatively modulated *SORBS1*.

### miR-223-3p targets *SORBS1 *to affect proliferation, migration and invasion of GC cells *SORBS1*

**Fig. 5:**
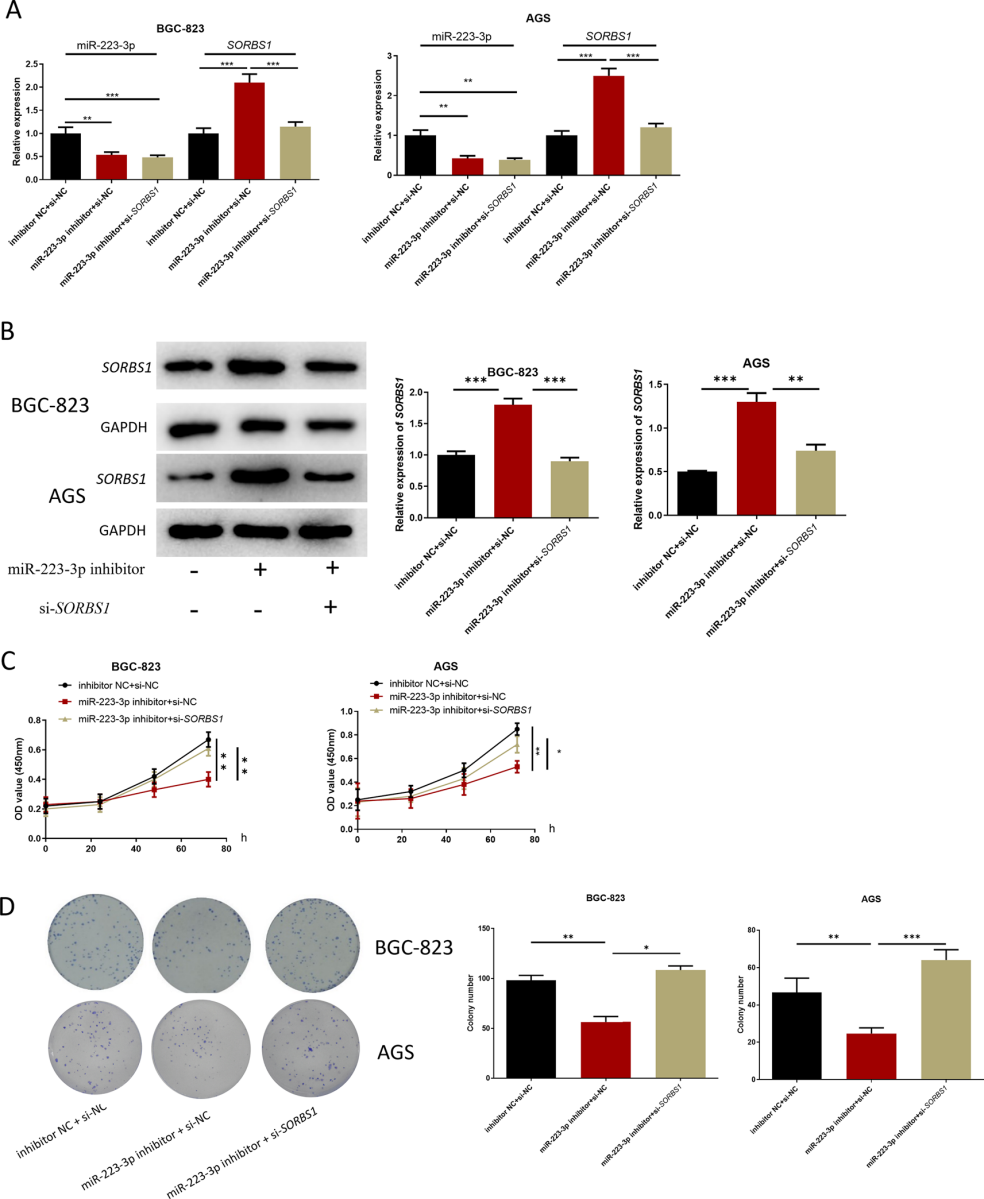

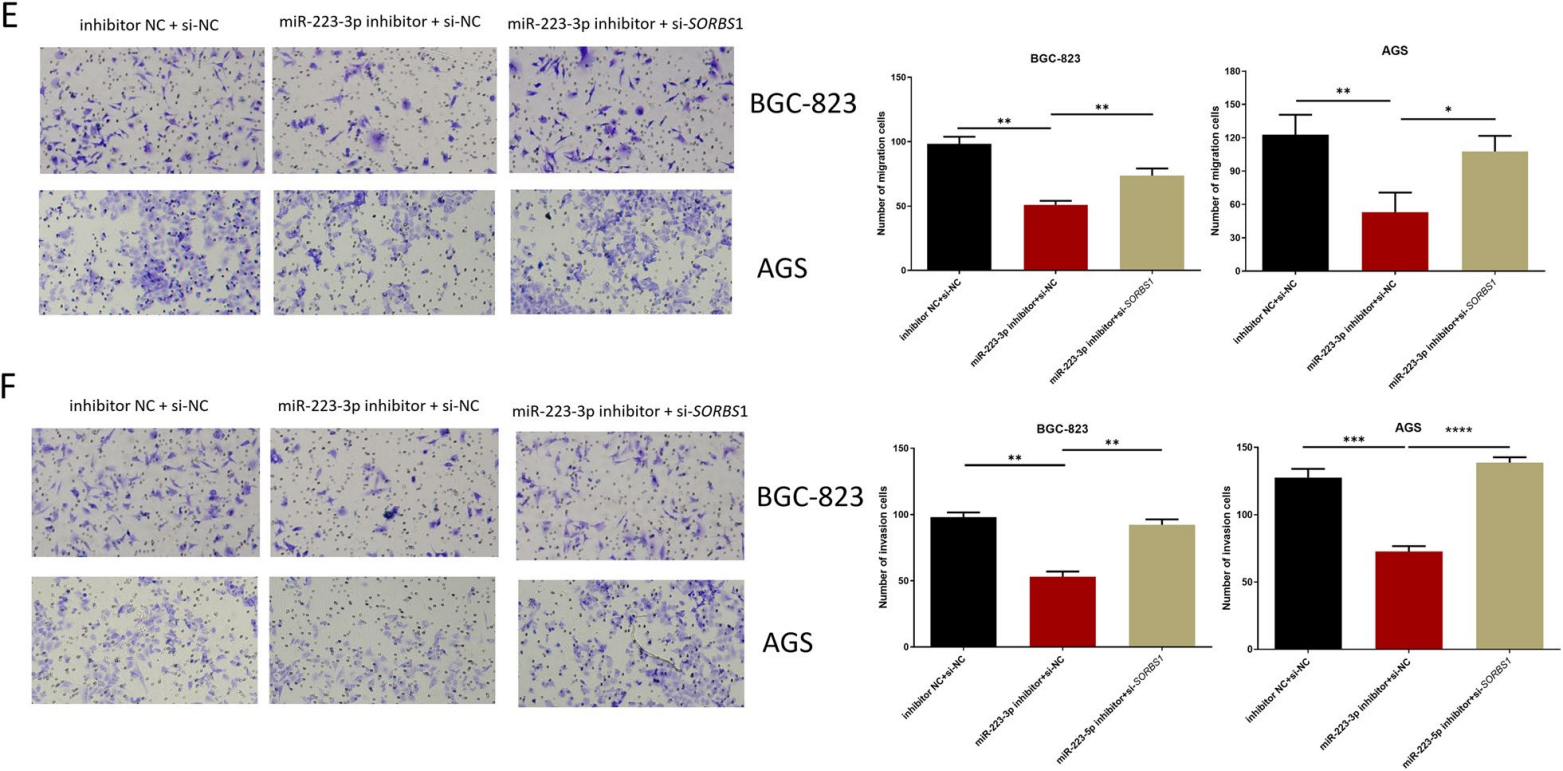

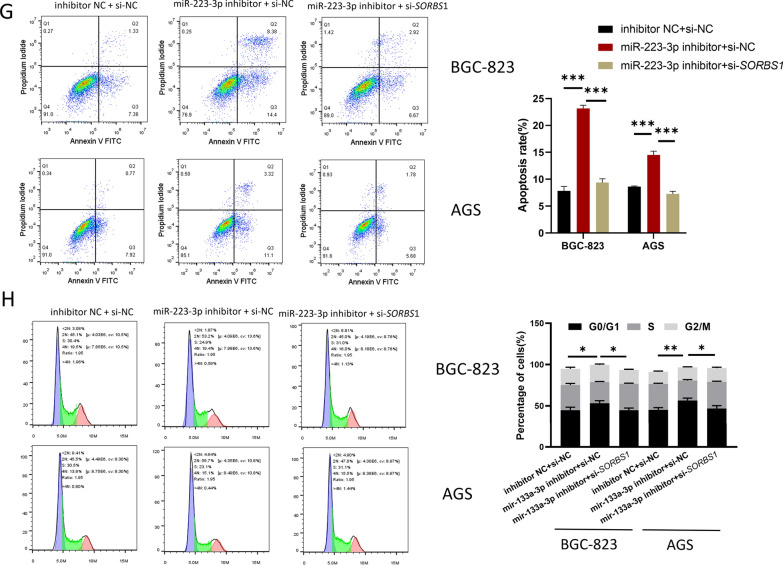
miR-223-3p regulates the proliferation, migration and invasion of GC cells by targeting *SORBS1. A* miR-223-3p and *SORBS1*expression in each transfection group after 48 h transfection (BGC-823 cells: n = 3; *p* = 0.0017; *p* < 0.001, *p* < 0.001; *p* < 0.001, AGS cells: *p* = 0.0025; *p* = 0.0017, *p* = 0.0003; *p* = 0.0004); **B** *SORBS1* expression in each transfection group after 48-h transfection (n = 3; *p* = 0.0003; *p* = 0.0002, n = 3; *p* = 0.0002; *p* = 0.0014); **C** The cell activity of each transfection group (n = 3; *p* = 0.0014; *p* = 0.0051, n = 3; *p* = 0.0014; *p* = 0.0187); **D** The ability of cell colony formation in each transfection group (n = 3; *p* = 0.0015; *p* = 0.0158, n = 3; *p* = 0.0098; *p* = 0.0004); **E**, **F** The cell migration and invasion in each transfection group (migration: n = 3; *p* = 0.0085; *p* = 0.0073, n = 3; *p* = 0088; *p* = 0.0138, invasion: n = 3; *p* = 0.0073; *p* = 0.0093, n = 3; *p* = 0.0002; *p* < 0.0001). **G**, **H** Cell apoptosis and cell cycle in each transfection group (apoptosis: n = 3; *p* < 0.0001; *p* < 0.0001, *p* = 0.0001; *p* = 0.0001, cell cycle: n = 3; *p* = 0.0370; *p* = 0.0225, *p* = 0.0085; *p* = 0.0217). *p < 0.05; **p < 0.01; ***p < 0.001

After GC cells were transfected with inhibitor NC + si-NC, miR-223-3p inhibitor + si-NC, miR-223-3p inhibitor + si-*SORBS1*, respectively, expression levels detection, and cell function tests were carried out on the co-transfected cells. qRT-PCR result displayed that in comparison with the inhibitor NC + si-NC group, miR-223-3p expression level in the miR-223-3p inhibitor + si-NC and miR-223-3p inhibitor + si-*SORBS1* groups was remarkably downregulated. Compared with inhibitor NC + si-NC group, *SORBS1* expression in miR-223-3p inhibitor + si-NC group was markedly upregulated, and restored in miR-223-3p inhibitor + si-*SORBS1* group (Fig. 5A). Western blot results were consistent with qRT-PCR result (Fig. 5B). CCK-8 and colony formation assays expressed that miR-223-3p inhibitor could inhibit the proliferation of GC cells, while si-*SORBS1* could reverse this inhibitory effect (Fig. 5C, D). The results of transwell migration and invasion assays showed that miR-223-3p inhibitor could restrain the migration and invasion of GC cells, and si-*SORBS1* could reverse this inhibitory effect (Fig. 5E, F). Flow cytometry result indicated that miR-223-3p inhibitor stimulated GC cell apoptosis and increased cells in G0/G1, whereas such effects were attenuated by si-*SORBS1* (Fig. 5G, H). Taken together, miR-223-3p regulated the progression of GC cells by mediating *SORBS1*.

### miR-223-3p in MVs derived from CAFs facilitates the malignant progression of GC in vivo

**Fig. 6:**
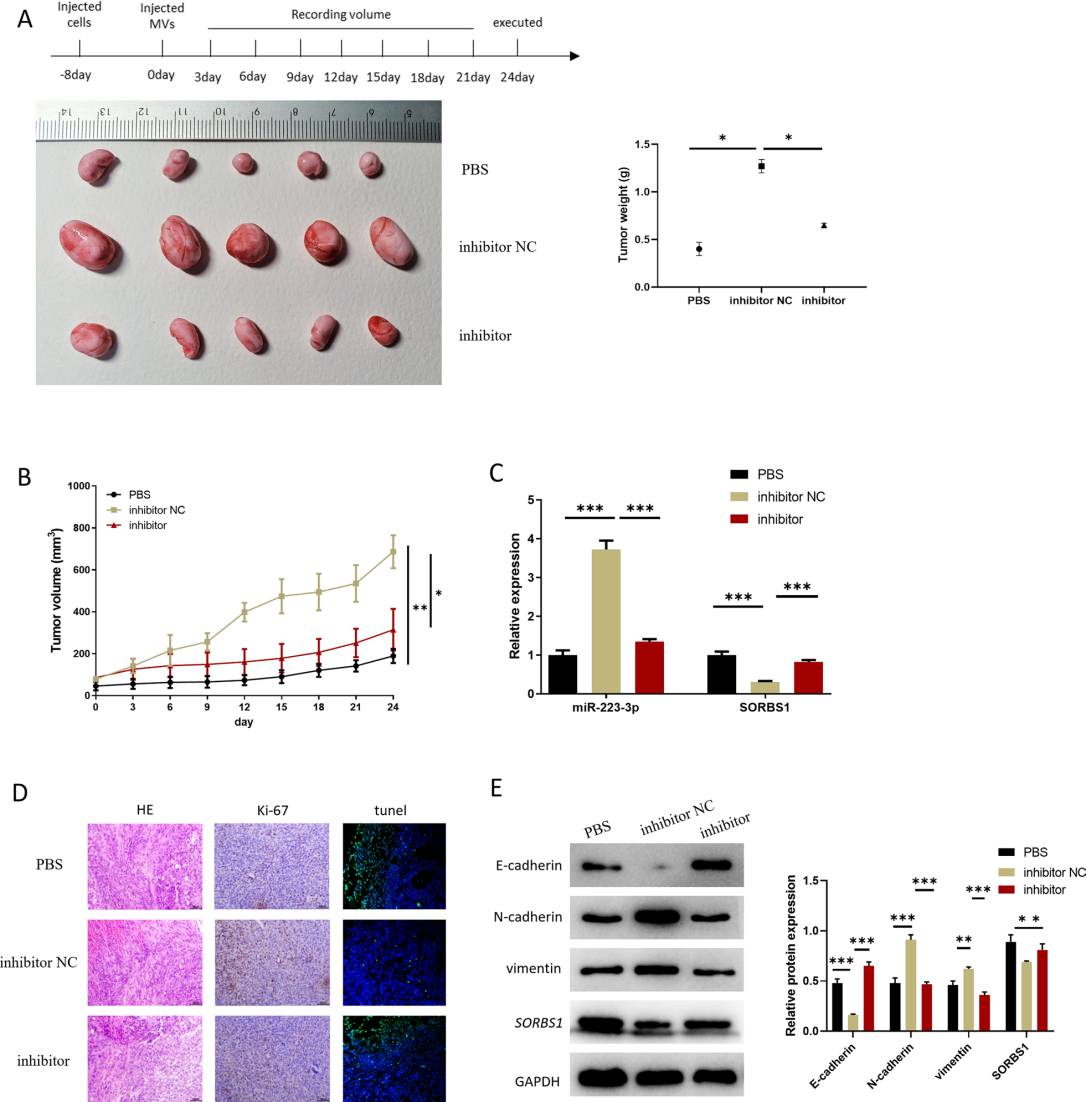
miR-223-3p in MVs derived from CAFs promotes malignant progression of GC in vivo. **A** Appearance pictures and weight of subcutaneous tumor masses of mice (n = 5; *p* < 0.0001; *p* < 0.0001); **B** Volume and growth of subcutaneous tumor masses of transplanted mice in the 3 groups (n = 5; *p* = 0.0013; *p* = 0.0196); **C** miR-223-3p and *SORBS1*levels in cancer tissue of 3 groups of mice (n = 3; *p* < 0.0001; *p* ≤ 0.0001, *p* = 0.0002; *p* = 0.0001); **D** Expression level of Ki-67 in tumor tissue of transplanted mice detected by IHC; **E** The expression levels of EMT-related proteins and *SORBS1* in the tumor tissue of transplanted mice (n = 3; *p* = 0.0002; *p* ≤ 0.0001, *p* = 0.0005; *p* = 0.0001, *p* = 0.0034; *p* = 0.0002). *p < 0.05; **p < 0.01; ***p < 0.001

GC cells were utilized to construct xenograft mouse models. Then CAFs-MVs (MVs isolated after CAFs were treated with miR-223-3p inhibitor and NC) and an equivalent volume of PBS was used to treat the tumor. Based on the above models, we conducted statistics on the weight and growth rate of tumor masses, finding that MVs hampered tumor mass growth and weight in CAFs after miR-223-3p knockdown (Fig. 6A, B). qRT-PCR result revealed that miR-223-3p level was reduced while *SORBS1* was elevated in the inhibitor group (Fig. 6C). In addition, IHC analysis revealed that the inhibitor group presented a smaller Ki-67 cell positive rate and more apoptotic cells compared with the inhibitor NC group (Fig. 6D). Finally, the expression of EMT-related proteins and *SORBS1* in tumor mass was detected by western blot (Fig. 6E). The result indicated that compared with the inhibitor NC group, *SORBS1* protein expression was upregulated and the EMT process of GC cells was prominently decreased in the inhibitor group. The above findings suggested that miR-223-3p carried by CAFs-derived MVs could boost the malignant progression of GC in vivo.

## Discussion

GC is caused by a variety of factors, including helicobacter pylori infection, smoking, dietary habits, and genetic factors [1]. Though the molecular mechanism of GC is not yet clear, a recent study showed that CAFs is crucial in promoting the development of GC, and its main mechanism is to secrete various growth factors, cytokines and RNA to promote the malignant progression of GC tissue, such as angiogenesis, drug resistance, proliferation, migration and invasion [3]. In the above processes, MVs often act as carriers to maintain the interaction between CAFs and cancer cells by transmitting signaling molecules [18]. Herein, it was uncovered that the MVs secreted by CAFs carried miR-223-3p and delivered it into GC cells, and accelerated the malignant progression of GC cells by mediating *SORBS1*.

miR-223-3p was highly expressed in GC in the previous bioinformatics analysis and the expression detection. This result is consistent with several published studies [11, 19, 20]. For example, Yiping Zhu et al. [11] found that compared with PT, miR-223-3p expression is high in GC tissue, and miR-223-3p level in GC tissue is positively correlated with lymph node metastasis and invasion depth. Similarly, it was discovered here that miR-223-3p was relatively highly expressed in GC tissue or GC cell lines.

At present, MVs have not been fully studied in GC, but some studies showed that specific MVs can influence GC or can be used as markers of GC [21–23]. Malgorzata Stec et al*.* [23] demonstrated that MVs derived from tumors can deliver signaling molecules to GC cells and promote tumor growth. Similar experimental results were also obtained in our study, but the difference was that the MVs in our study were derived from CAFs. Hence, it could be seen that there may be different sources of MVs in GC tissue that simultaneously affected the behavior and function of GC cells.

In fact, there are few studies on *SORBS1* in cancer, and no studies have illustrated *SORBS1* expression in GC and its mechanism. However, studies related to breast cancer showed that *SORBS1* is lowly expressed in breast cancer patients [24], and forced expression of *SORBS1* can suppress the metastasis of cancer cells and improve the sensitivity of cancer cells to chemotherapy drugs [15]. Similarly, it was noted from our experimental results that *SORBS1* was less expressed in GC cell lines. miR-223-3p regulated the progression of GC cells by targeting *SORBS1*. Hence, the role of *SORBS1* in GC is similar to that in breast cancer. Down-regulating *SORBS1* by miR-223-3p with high expression in CAFS-MVs may provide a mechanism for promoting the development of GC, but it is by no means the only one. miR-223-3p can accelerate GC cell processes by regulating multiple target genes and pathway proteins (Arid1a, NLRP3, and NDRG1). Whether other genes and pathways are implicated in GC biological functions requires further research.

Taken together, our study indicated that CAFs-derived MVs carried miR-223-3p and delivered it into GC, and targeted *SORBS1* to boost the cell proliferation, migration, invasion, and EMT process, and modulate cell apoptosis and cell cycle in GC. The study of this mechanism also offers a novel theoretical basis for GC diagnosis and therapy.

## Data Availability

The data and materials in the current study are available from the corresponding author on reasonable request.
