# Correction to: miR-223-3p carried by cancer-associated fibroblast microvesicles targets SORBS1 to modulate the progression of gastric cancer

**DOI:** 10.1186/s12935-022-02571-5

**Published:** 2022-04-20

**Authors:** Xiaoli Jin, Xi Qiu, Yi Huang, Hang Zhang, Kaibo Chen

**Affiliations:** 1 Department of Gastrointestinal Surgery, The Second Affiliated Hospital of Zhejiang University School of Medicine, 88 Jiefang Road, Hangzhou, 310009 Zhejiang China; 2 Department of Hematology, The Second Affiliated Hospital of Zhejiang University School of Medicine, Hangzhou, 310009 Zhejiang China

## Correction to: Cancer Cell International (2022) 22:96 https://doi.org/10.1186/s12935-022-02513-1

In this article [1], the data and p value of the Western Blot histogram were found. Due to the software operation error, a calculation error occurred. After repeated quantitative review several times in the proofing review stage, we reconfirmed the gray value of the Western Blot band, so modified. The Western Blot histogram in the picture and the p value marked in the legend part of the Figure legends, the corrected Figs. 2, 3, 4, 5 and 6 and its legends are given in this correction.

**Fig. 2:**
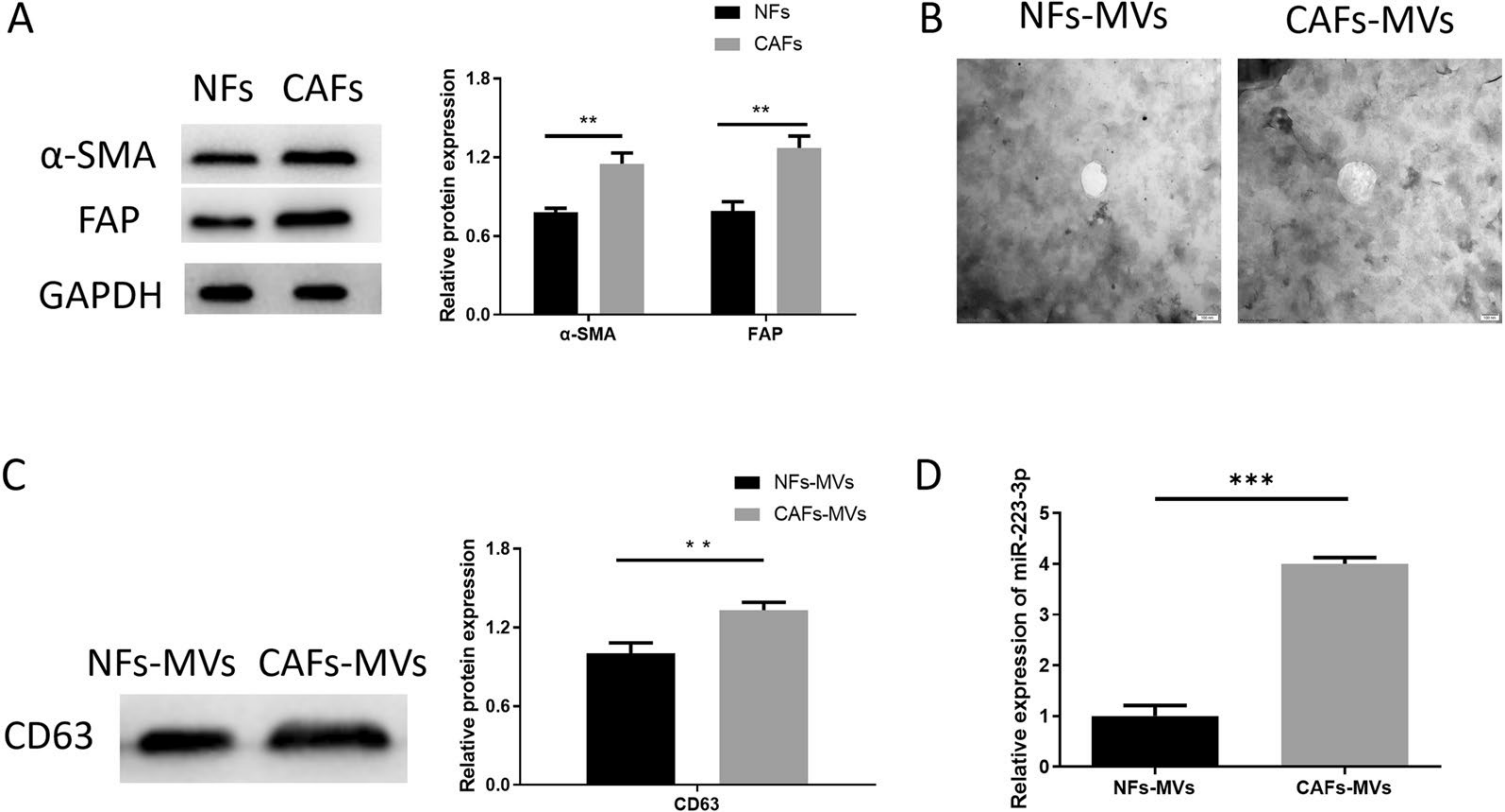
miR-223-3p expression is upregulated in MVs derived from CAFs. **A** Detection of fibroblast marker proteins (α-SMA, FAP) and GC markers (CEA, CK-18) in CAFs, NFs (at logarithmic phase) by Western Blot (n = 3, *p* = 0.0017; *p* = 0.0019); **B** NFs-MVs and CAFs-MVs observed under TEM with a scale of 100 nm; **C** MV marker protein CD63 in NFs-MVs and CAFs-MVs detected by Western Blot (n = 3, *p* = 0.0046); **D** miR-223-3p expression in CAFs-MVs and NFs-MVs (n = 3, *p* < 0.001). *p < 0.05; **p < 0.01; ***p < 0.001

**Fig. 3:**
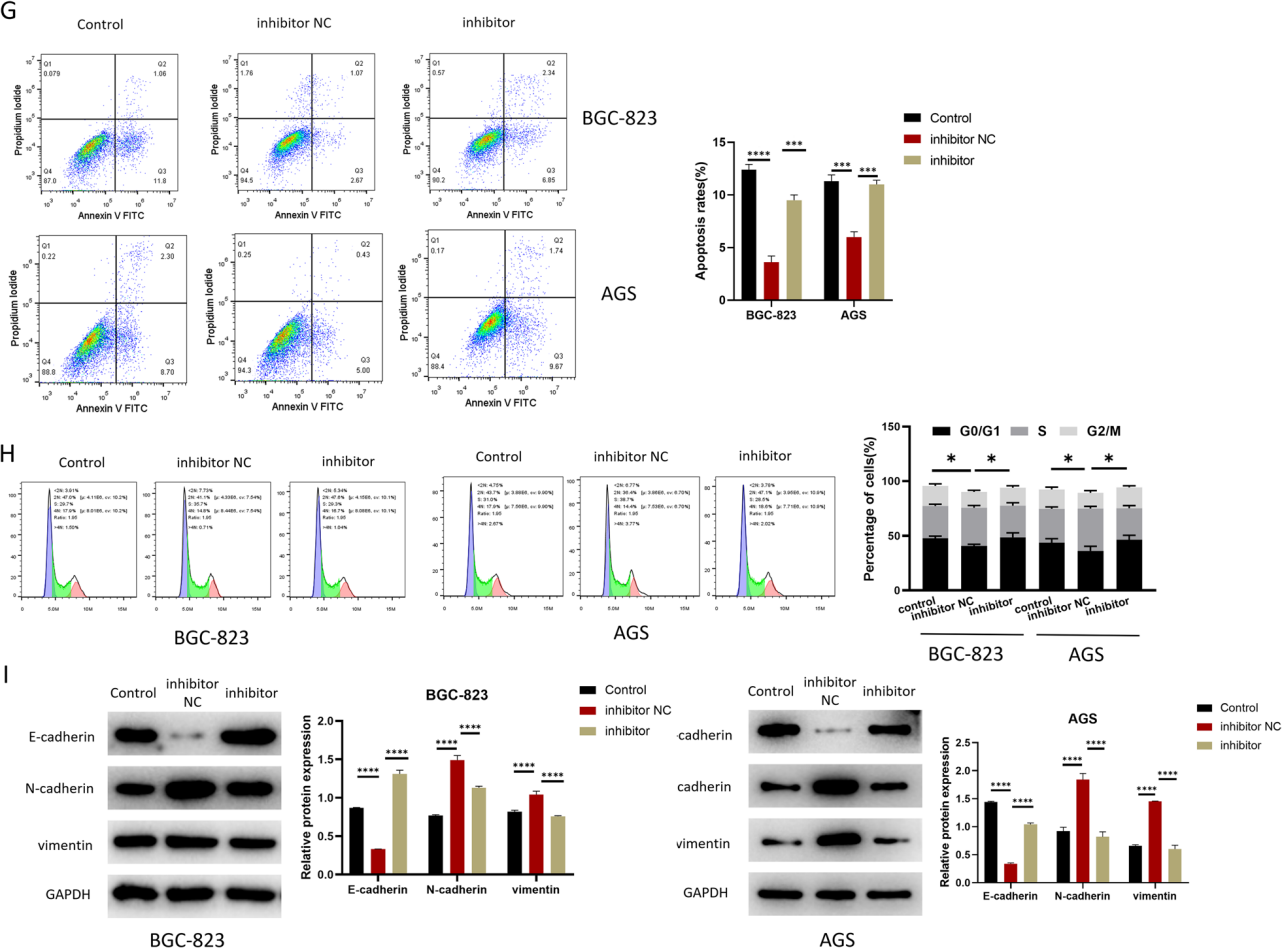
CAFs-MVs-derived miR-223-3p can facilitate malignant progression of GC. **A** PKH26-labeled MVs co-incubated with GC cells (PKH26 in red while DAPI in blue) (n = 3, *p* < 0.0001; *p* < 0.001; scale bar 20 μm); **B** miR-223-3p level in the CAFs (at logarithmic phase, n = 3, *p* = 0.0237) and MVs of CAFs (n = 3, *p* = 0.0021) in each transfection group (inhibitor NC, inhibitor); **C** miR-223-3p level in GC cells after 24 h of MVs co-cultured with GC cells (n = 3; *p* < 0.001; *p* < 0.001, n = 3; *p* = 0.0003; *p* = 0.0006); **D** the activity of GC cells after co-culture with MVs in each transfected group (control, inhibitor NC, inhibitor) (n = 3; *p* < 0.001; *p* = 0.0028, n = 3; *p* = 0.0011; *p* = 0.0214); **E** the colony-forming ability of GC cells after co-culture with MVs in each transfection group (n = 3; *p* = 0.0125; *p* = 0.0459, n = 3; *p* = 0.0062; *p* = 0.0023); **F** the migration and invasion of GC cells after co-culture with MVs in each transfected group (BGC-823 cells: n = 3; *p* < 0.001; *p* < 0.001, n = 3; *p* = 0.0001; *p* = 0.0003, AGS cells: n = 3; *p* = 0.0001; *p* < 0.0001, n = 3; *p* = 0.0015; *p* = 0.0007); **G** Cell apoptosis status (n = 3; *p* < 0.0001; *p* = 0.0002, n = 3; *p* = 0.0003; *p* = 0.0002); **H** cell cycle status (n = 3; *p* = 0.0106; *p* = 0.0350, n = 3; *p* = 0.0101; *p* = 0.0224); **I** the expression of EMT-related proteins in GC cells after co-culture with MVs 24 h in each transfected group (BGC-823 cells: n = 3; *p* < 0.0001; *p* < 0.0001, *p* < 0.0001; *p* < 0.0001, *p* < 0.0001; *p* < 0.0001, AGS cells: *p* < 0.0001; *p* < 0.0001, *p* < 0.0001; *p* < 0.0001, *p* < 0.0001; *p* < 0.0001). *p < 0.05; **p < 0.01; ***p < 0.001

**Fig. 4:**
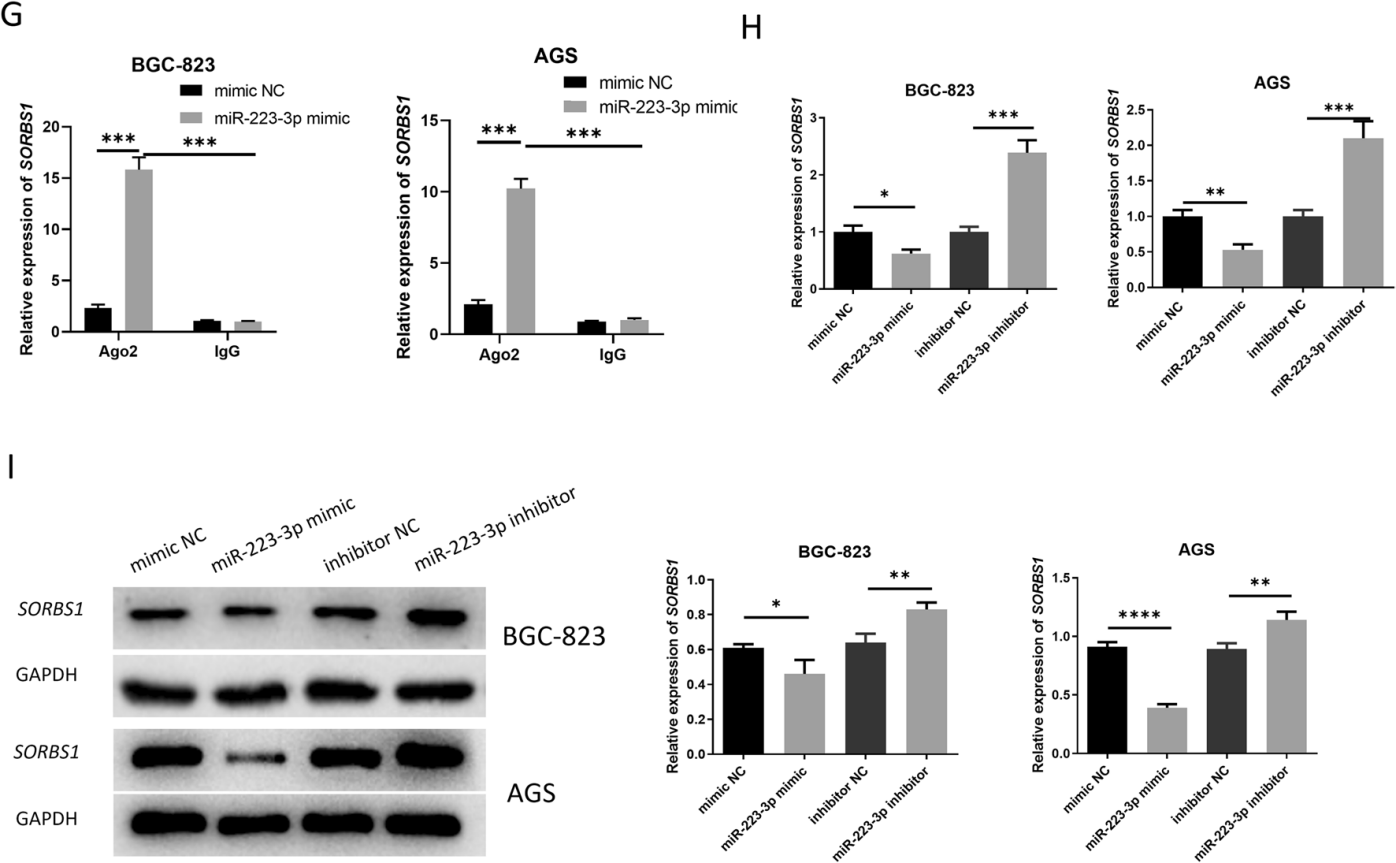
miR-223-3p targets and modulates *SORBS1.* **A** Differential expression of GC-related mRNA by analysis in TCGA-STAD; **B** the intersection between results predicted by 3 databases (Targetscan, miRDB, mirDIP) and the differential genes; **C** the correlation between miR-223-3p and potential target genes analyzed by ENCORI (*p* = 2.41e−19); **D** *SORBS1* expression in normal gastric mucosa cells and GC cells (atlogarithmic phase) (n = 3; *p* = 0.0002; *p* < 0.0001; *p* = 0.001); **E** the binding sites of miR-223-3p and *SORBS1* predicted by miRDB database; **F** Targeted relationship between miR-223-3p and *SORBS1* verified by dual-luciferase reporter gene detection (n = 3; *p* = 0.0092); **G** Interaction between miR-223-3p and *SORBS1* verified by RIP (n = 3; *p* < 0.0001; *p* < 0.0001, n = 3; *p* < 0.0001; *p* < 0.0001); **H** *SORBS1* level in each transfection group after 48-h transfection (n = 3; *p* = 0.0367; *p* < 0.001, n = 3; *p* = 0.0024; *p* = 0.0017); **I** the protein expression level of *SORBS1* in each transfection group after 48-h transfection (n = 3; *p* = 0.0345; *p* = 0.0068, n = 3; *p* < 0.0001; *p* = 0.0073). *p < 0.05; **p < 0.01; ***p < 0.001

**Fig. 5:**
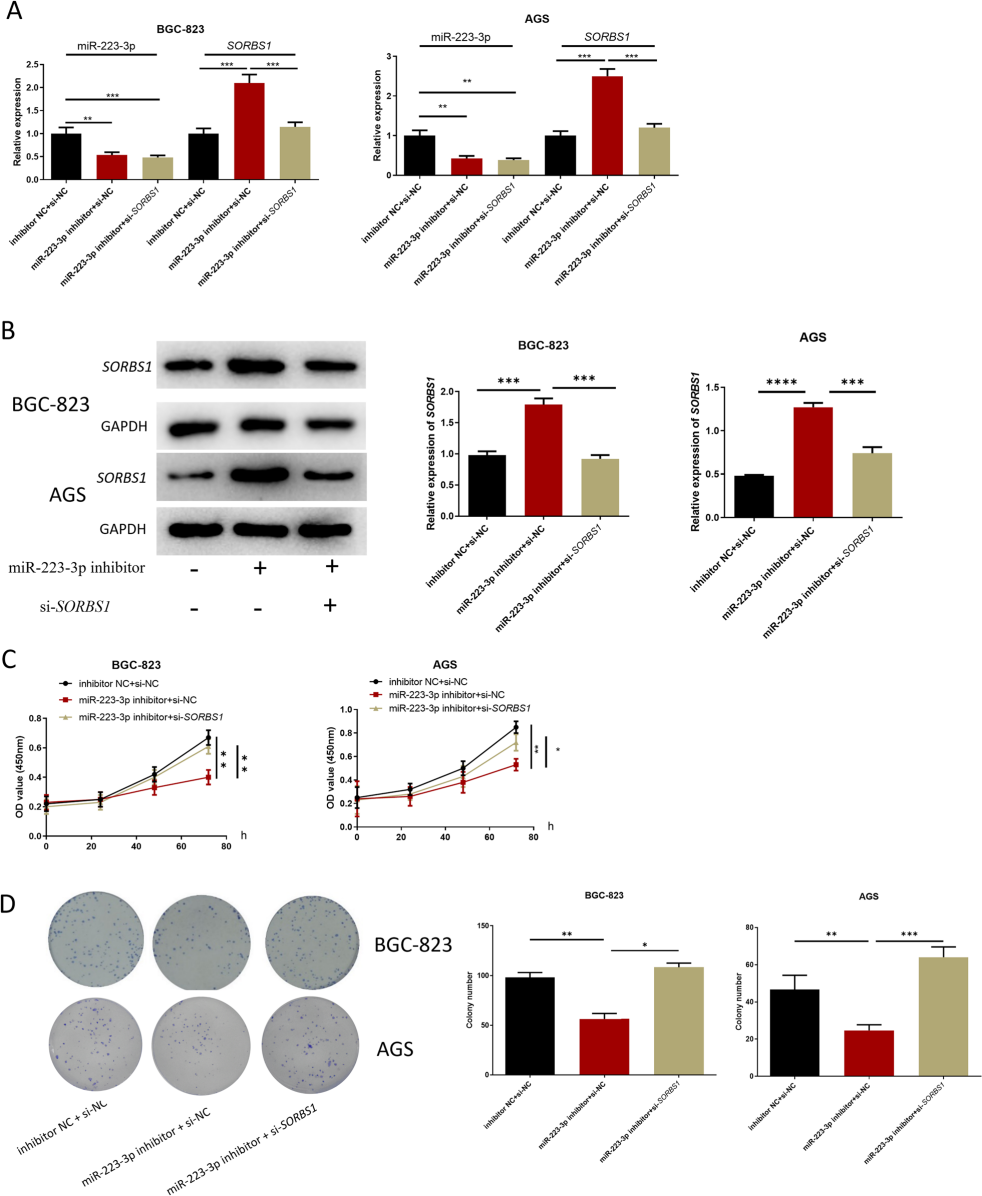
miR-223-3p regulates the proliferation, migration and invasion of GC cells by targeting *SORBS1.* **A** miR-223-3p and *SORBS1*expression in each transfection group after 48-h transfection (BGC-823 cells: n = 3; *p* = 0.0017; *p* < 0.001, *p* < 0.001; *p* < 0.001, AGS cells: *p* = 0.0025; *p* = 0.0017, *p* = 0.0003; *p* = 0.0004); **B** *SORBS1* expression in each transfection group after 48-h transfection (n = 3; *p* = 0.0003; *p* = 0.0002, n = 3; *p* < 0.001; *p* = 0.0004); **C** the cell activity of each transfection group (n = 3; *p* = 0.0014; *p* = 0.0051, n = 3; *p* = 0.0014; *p* = 0.0187); **D** the ability of cell colony formation in each transfection group (n = 3; *p* = 0.0015; *p* = 0.0158, n = 3; *p* = 0.0098; *p* = 0.0004); **E**, **F** the cell migration and invasion in each transfection group (migration: n = 3; *p* = 0.0085; *p* = 0.0073, n = 3; *p* = 0088; *p* = 0.0138, invasion: n = 3; *p* = 0.0073; *p* = 0.0093, n = 3; *p* = 0.0002; *p* < 0.0001). **G**, **H** Cell apoptosis and cellcycle (apoptosis: n = 3; *p* < 0.0001; *p* < 0.0001, *p* = 0.0001; *p* = 0.0001, cell cycle: n = 3; *p* = 0.0370; *p* = 0.0225, *p* = 0.0085; *p* = 0.0217). *p < 0.05; **p < 0.01; ***p < 0.001

**Fig. 6:**
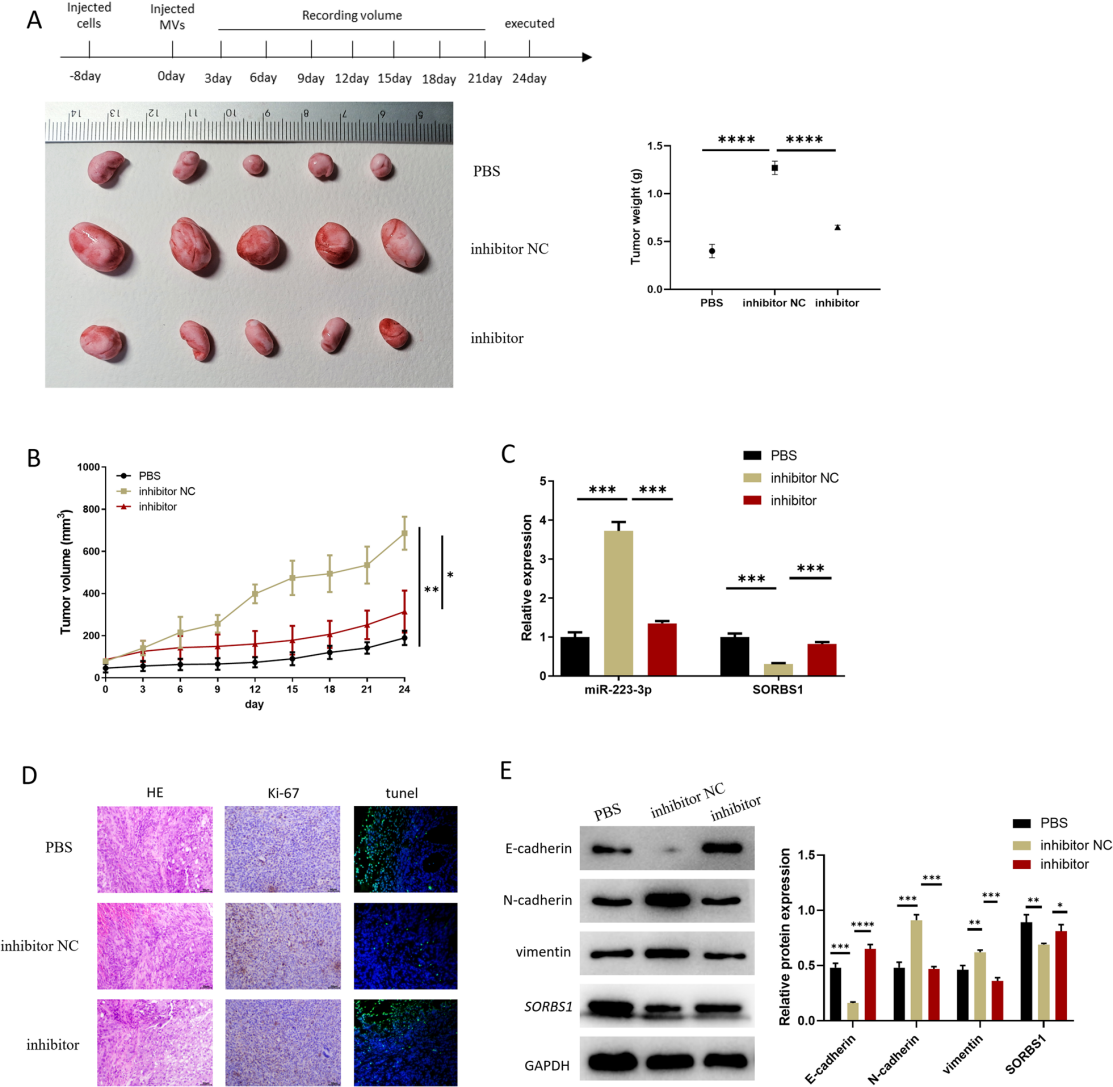
miR-223-3p in MVs derived from CAFs promotes malignant progression of GC in vivo. **A** Appearance pictures and weight of subcutaneous tumor masses of mice (n = 5; *p* < 0.0001; *p* < 0.0001); **B** volume and growth of subcutaneous tumor masses of transplanted mice in the 3 groups (n = 5; *p* = 0.0013; *p* = 0.0196); **C** miR-223-3p and *SORBS1*levels in cancer tissue of 3 groups of mice (n = 3; *p* < 0.0001; *p* ≤ 0.0001, *p* = 0.0002; *p* = 0.0001); **D** expression level of Ki-67 in tumor tissue of transplanted mice detected by IHC; **E** the expression levels of EMT-related proteins and *SORBS1* in the tumor tissue of transplanted mice (n = 3; *p* = 0.0002, *p* ≤ 0.0001; *p* = 0.0005, *p* = 0.0001; *p* = 0.0034,*p* = 0.0002; *p* = 0.008, *p* = 0.0269). *p < 0.05; **p < 0.01; ***p < 0.001
